# Reliability and validity of the fixed-frame portable dynamometer in assessing ankle force sense in individuals with and without chronic ankle instability

**DOI:** 10.1371/journal.pone.0345162

**Published:** 2026-03-19

**Authors:** Jianglong Zhan, Peng Chen, Zhongqi Yu, Teck Cheng Tan, Chaoyu Guo, Menghan Xu, Van Minh Le, Lin Wang

**Affiliations:** 1 School of Exercise and Health, Shanghai University of Sport, Shanghai, China; 2 Sports Medicine and Rehabilitation Center, Shanghai University of Sport, Shanghai, China; 3 Shanghai Shangti Orthopaedic Hospital, Shanghai, China; 4 Shanghai Warrior Shoes Co., Ltd., Shanghai, China; Università degli Studi di Milano: Universita degli Studi di Milano, ITALY

## Abstract

**Background:**

Deficits in sagittal-plane ankle force sense impair the ankle strategy for counteracting perturbations and are associated with balance impairments in individuals with chronic ankle instability (CAI). However, standardized assessment devices, such as the CON-TREX dynamometry system, are expensive and impractical. In this study, a fixed-frame portable dynamometer (FF-PD) was developed, and its concurrent validity, discriminant validity, and test–retest inter-session reliability were evaluated in measuring ankle plantarflexion and dorsiflexion force sense in individuals with CAI, copers, and healthy controls.

**Methods:**

A total of 72 participants (24 with CAI, 24 copers, and 24 healthy controls) performed force-matching tests utilizing the FF-PD and CON-TREX, and the FF-PD retested one week later. Concurrent validity was evaluated with Pearson correlations; discriminant validity, with one-way ANOVA and receiver operating characteristic (ROC) analyses; and reliability, with intraclass correlation coefficients (ICC), standard error of measurement (SEM), and minimal detectable change at the 95% confidence level (MDC_95_).

**Results:**

FF-PD exhibited moderate-to-strong correlations with CON-TREX (*r* = 0.597–0.770, *p* ≤ 0.002). For discriminant validity, the FF-PD effectively distinguished individuals with CAI from healthy controls in plantarflexion (*p* = 0.011) and dorsiflexion (*p* = 0.034). ROC-derived cutoffs were 12.06% for plantarflexion and 9.57% for dorsiflexion. Test–retest inter-session reliability was good-to-excellent (ICC = 0.794–0.960), with low SEM (0.9%–2.6%) and clinically meaningful MDC_95_ (2.5%–7.4%).

**Conclusion:**

The FF-PD is a valid and reliable device for assessing ankle force sense in plantarflexion and dorsiflexion. It effectively differentiates individuals with CAI from healthy controls, and ROC-derived cutoff values provide clinically interpretable thresholds that may support clinical screening and decision-making, as well as the potential use in monitoring changes during clinical screening and rehabilitation.

## Introduction

Maintaining an upright stance and balance relies on several motor strategies, among which the ankle strategy is the most fundamental [1]. By adjusting plantarflexion and dorsiflexion in the sagittal plane, this strategy helps regulate the body’s center of mass and counteract mild-to-moderate perturbations [1]. This process relies not only on muscular performance but also on proprioceptive input, particularly force sense, which is pivotal for perceiving and controlling forces [2,3]. Impairment of force sense can compromise the fine-tuning of muscle contractions, thereby reducing postural control [4]. Such deficits are observed not only in individuals recovering from sports injuries but also in the elderly and patients undergoing neurological rehabilitation [5–7]. Thus, assessing sagittal-plane force sense holds significant value for postural control research and clinical rehabilitation applications.

The reliable evaluation of force sense has typically been conducted using standardized dynamometry systems, such as the CON-TREX system, which has been extensively employed in CAI research [5,8,9]. However, these systems are expensive and dependent on laboratory settings due to large space requirements, limited portability, and the need for extensive examiner training, which limits their practicality for routine screening, rehabilitation monitoring, and field applications. Owing to advancements in technology, portable devices (such as dynamometers and electrogoniometers) have been increasingly used for the assessment of kinetics and joint kinematics, offering advantages such as portability, tablet integration, and real-time feedback, while demonstrating good-to-excellent reliability and validity [10–13]. Nevertheless, their handheld operation renders them unsuitable for evaluating force sense (i.e., force-matching tests), as stability and repeatability during assessment cannot be guaranteed. Consequently, the reliability and validity of portable dynamometers for force sense assessment remain insufficiently established. To address this limitation, we developed a fixed frame that integrates with the portable dynamometer to evaluate force sense during ankle plantarflexion and dorsiflexion.

The occurrence of ankle sprains and their progression to chronic ankle instability (CAI) are primarily attributed to impaired ankle stability, which depends heavily on force sense [4,14,15]. Research further suggests that deficits in ankle force sense are strongly linked to balance impairments in individuals with CAI, with sagittal-plane deficits appearing as a particularly sensitive indicator of this dysfunction [5]. Therefore, this study, using CAI as a representative condition, aimed to: (1) assess the concurrent validity of a fixed-frame portable dynamometer (FF-PD) as compared with the CON-TREX across various populations, including individuals with CAI, copers (those with a history of ankle sprain but without instability), and healthy controls; and (2) evaluate the test–retest inter-session reliability and discriminative validity of the FF-PD within these populations. We posited that the force-matching performance in FF-PD: (1) would exhibit a moderate correlation with that of the CON-TREX; (2) would show robust test–retest inter-session reliability; and (3) would effectively differentiate between CAI and healthy controls.

## Methods

The sample size was established based on guidelines for reliability and validity research. Sample size for test–retest reliability was estimated using the method proposed by Walter et al. for intraclass correlation coefficients [16]. The calculation assumed a minimally acceptable ICC (ICC_0_) of 0.70, an expected ICC (ICC_1_) of 0.85, a significance level of α  =  0.05, statistical power of 0.80, and two repeated measurements (k  =  2). Based on these assumptions, a minimum of 43 participants was required. To account for a potential 5% drop-out between sessions, a total of 46 participants were recruited. Furthermore, given that this study aimed to determine whether the developed device can differentiate among the three groups, we performed an a priori sample size estimation with one-way ANOVA, which was implemented in G*Power (version 3.1.9.2). With an α value of 0.05, an effect size of 0.375 derived from previously reported dorsiflexion force sense differences between individuals with CAI and healthy controls [5], and a power of 0.80, the minimum sample size was determined to be 72 participants.

All participants were aged 18–30 years and were right-leg dominant (defined as the leg used for kicking [17]). Restricting inclusion to the dominant leg was intended to reduce potential laterality-related variability in sensorimotor control and to standardize testing conditions for this first validation study. We adhered to the participant selection criteria established by the International Ankle Consortium for CAI and the recommendations for identifying copers [18]. Eligibility for both the CAI and coper groups necessitated a history of at least one right ankle sprain occurring more than 12 months prior, accompanied by swelling and pain that restricted daily activities for a minimum of one day. Furthermore, the participants with CAI were required to report (1) a minimum of two episodes of ankle instability within the past six months and (2) a Cumberland Ankle Instability Tool (CAIT) score of ≤ 24 [19], whereas the copers were required to report (1) no more than one instability episode within the past six months and (2) a CAIT score of ≥ 28 [20]. The eligibility criteria for the healthy group were as follows: (1) no history of ankle sprain; and (2) a CAIT score of 30 [19]. The participants were excluded if they had a history of neuromusculoskeletal disorders or any conditions linked to sensorimotor dysfunction. The recruitment period was from 16/02/2025–20/05/2025. This study was approved by the ethics committee of Shanghai University of Sport (approval number: 102772024RT162), and written informed consent was obtained from all participants before data collection.

### Instruments

**Fig 1:**
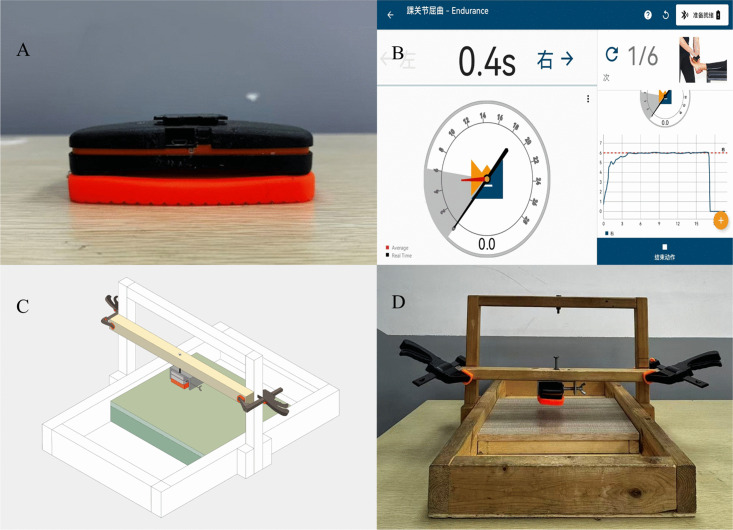
A fixed-frame portable dynamometer (FF-PD) for ankle force sense assessment. **(A)** Portable dynamometer. **(B)** Visual feedback interface of the force-matching test. **(C)** Design diagram of the device. **(D)** Actual photograph of the device.

The FF-PD, a custom-built device, was developed to perform stationary force-matching tasks (Fig 1C and 1D). The apparatus comprised three primary components: (1) a fixed wooden support frame consisting of a base plate, two vertical posts, and a top crossbar; (2) a movable supporting platform; and (3) an adjustable force-measurement module. The module comprised a portable dynamometer (K-Force, Kinvent, France, Fig 1A), a detachable crossbar, stainless-steel clamps, bolts, and F-clamps. The dynamometer was fastened to the crossbar with stainless-steel clamps and bolts, and the crossbar was affixed to the vertical posts of the support frame with F-clamps.

### Measurement procedures

Each participant was assessed in two sessions separated by one week. In the first session, after demographic data collection and procedure familiarization, the participants performed right ankle plantarflexion and dorsiflexion force-matching tests using the FF-PD and CON-TREX (PM-MJ, Physiomed, Germany) devices in a randomized order, counterbalanced for device sequence and movement direction. In the second session, the participants completed the force-matching tests using the FF-PD alone.

In the force-matching tests, the participants performed three maximum voluntary isometric contraction (MVIC) trials for ankle plantarflexion and dorsiflexion after a warm-up, and 25% of the average value was set as the target force for each movement direction. Low-to-moderate submaximal target forces (particularly  ≤  30% MVIC), compared with maximal force production, place greater emphasis on sensorimotor control while minimizing fatigue [4,21,22]. As 25% MVIC is commonly used in force-matching paradigms involving individuals with CAI and healthy controls [3,5,23,24], it was selected to ensure consistency with prior studies and comparability of results. After a three-minute interval, the participants executed a single five-second practice trial with visual feedback, during which they were instructed to match the real-time force as closely as possible with the target level (Fig 1B) [3]. They were subsequently required to consistently reproduce the target force for 15 seconds without feedback [3]. Each direction was tested three times, and a 30-second rest was allowed between trials.

**Fig 2:**
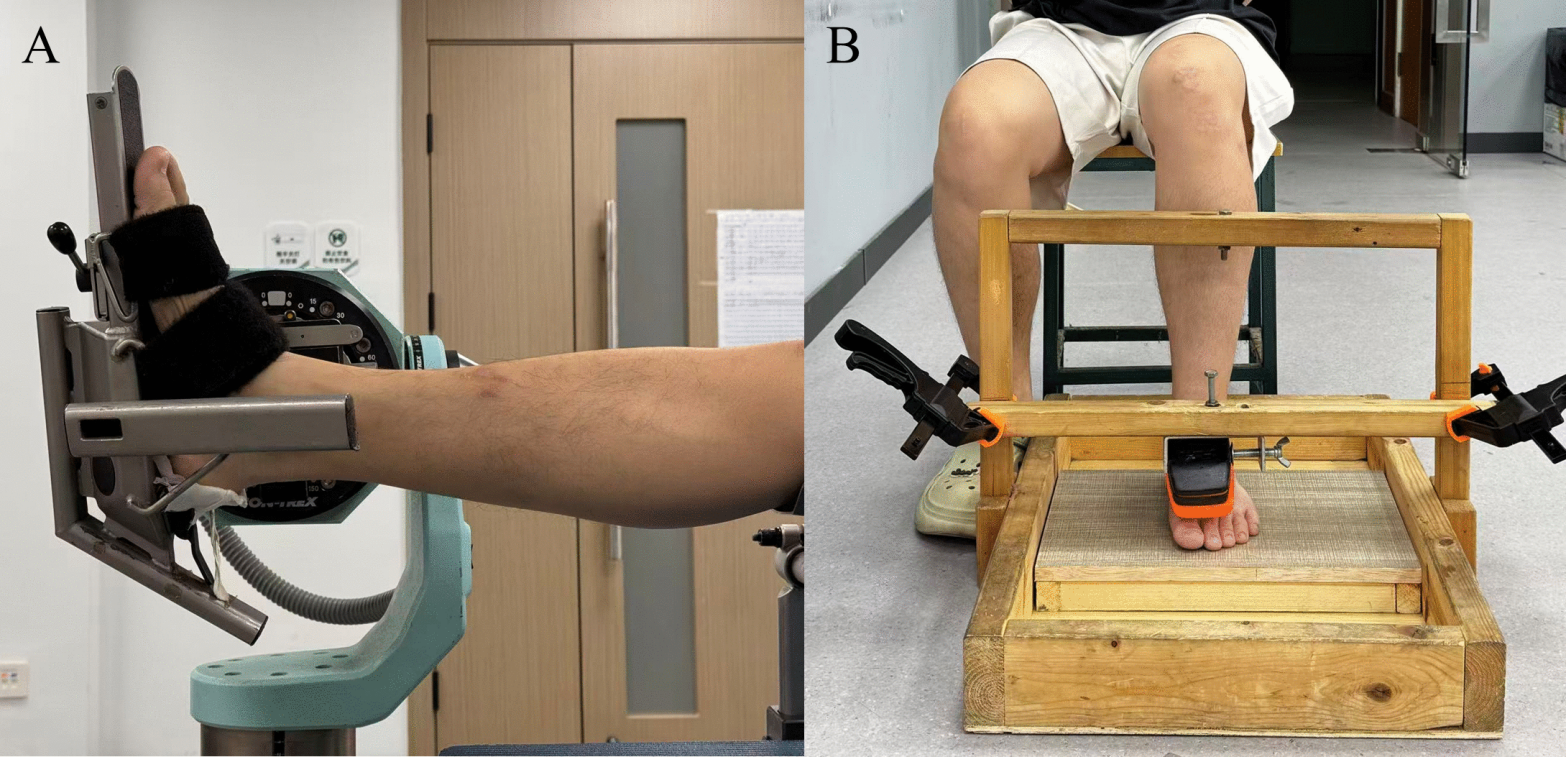
Experimental setup for ankle force sense assessment using the CON-TREX and the FF-PD. **(A)** Force sense assessment performed with the CON-TREX, with the participant positioned supine and the tested foot secured to the dynamometer footplate using straps. **(B)** Force sense assessment performed with the FF-PD, with the participant seated upright and the tested foot placed beneath the portable dynamometer, which was fixed within a custom-built wooden frame to ensure stability during force matching.

The test configurations varied among devices. During the FF-PD test, participants were seated with the hip, knee, and ankle joints maintained at 90°, and the right foot positioned beneath the portable dynamometer (Fig 2A). Participants were instructed to maintain an upright trunk posture and keep their arms crossed over the chest to minimize upper-body involvement or assistance. Real-time force data were transmitted via Bluetooth to a tablet (MediaPad M5 Lite, Huawei, China) running the KFORCE application (Kinvent, Montpellier, France), where the data were displayed and recorded. During the CON-TREX test, participants were positioned supine with the right foot secured to the dynamometer using straps (Fig 2B), while maintaining their arms crossed over the chest to restrict non-task-related movements. Force data were transmitted via cable to a laptop (ThinkPad, Lenovo, China) for visualization and recording.

The force-matching tests were supervised by a trained assessor according to standardized protocol, while another assessor, who was blinded to the group assignments and outcomes, operated the application. To maintain blinding, the two assessors were directed to refrain from discussing the test results.

Data from the force-matching tests were processed using MATLAB R2018b (MathWorks, Inc., Natick, USA). The mean value during feedback control was defined as the target force. The absolute error (AE) between the target force and the no-feedback force was subsequently computed and normalized to MVIC to produce AE%. The mean of three trials was utilized to assess ankle force sense, with smaller AE% indicating better force control.

### Statistical analysis

All statistical analyses were performed using SPSS 21.0 software (SPSS Inc., Chicago, IL, USA). The signifcance level was set at *p*  <  0.05 for all analyses. Continuous data were presented as mean  ±  standard deviation (SD). The Shapiro–Wilk test was conducted to evaluate the normality of the data.

To evaluate concurrent validity, Pearson correlation analysis was conducted to examine the association between AE% values obtained from the CON-TREX and the FF-PD devices across both individual groups and the overall sample. Correlation coefficients were interpreted in absolute terms as very weak (*r*  <  0.19), weak (*r*  =  0.20–0.39), moderate (*r*  =  0.40–0.59), strong (*r*  =  0.60–0.79), and very strong (*r*  >  0.80) [25].

To evaluate discriminant validity, one-way ANOVA was conducted to examine differences in AE% from the FF-PD force-matching test among the CAI, coper, and healthy groups. Significant effects were followed by post hoc comparisons, and effect sizes for pairwise differences were quantified using Cohen’s *d*. For group pairs showing significant differences, receiver operating characteristic analysis was used to determine cutoff AE%, with the optimal cutoff calculated as the maximum Youden index, representing the largest difference between sensitivity and 1 minus specificity [26].

To assess test–retest inter-session reliability, ICCs and Bland–Altman plots were employed. ICCs were computed using a two-way mixed-effects model with single measures and absolute agreement, evaluating the consistency of AE% from the FF-PD force-matching test across sessions. Agreement strength was categorized as excellent (>0.90), good (0.75–0.90), moderate (0.50–0.75), or poor (<0.50) [27]. The standard error of measurement (SEM) was calculated as the square root of the residual mean square derived from the ANOVA [28]. The minimal detectable change at the 95% confidence level (MDC_95_) was computed as 2.77 times the SEM, indicating the smallest change that can be considered genuine rather than a result of measurement error [29].

## Results

A total of 72 participants were recruited (24 with CAI, 24 copers, and 24 healthy controls) in this study. No demographic differences were found among the groups (*p*  >  0.05, Table 1). CAIT scores, however, were significantly lower in the CAI group than in the coper and healthy groups, and lower in the copers group than in the healthy controls (all *p*  <  0.001).

**Table 1:** Demographic characteristics and CAIT scores of the participants.

	CAI (*n* = 24)	Coper (*n* = 24)	Healthy (*n* = 24)
Age, year	21.0 ± 1.8	21.2 ± 3.0	21.2 ± 2.6
Height, m	1.75 ± 0.09	1.76 ± 0.07	1.74 ± 0.08
Weight, kg	70.4 ± 10.0	70.3 ± 9.2	69.1 ± 8.7
BMI, kg/m^2^	22.9 ± 1.9	22.7 ± 2.2	22.9 ± 2.9
Male/Female	17/7	18/6	17/7
CAIT score	15.6 ± 4.0	28.2 ± 0.4^#^	30^*^^✝^

Notes: BMI: Body mass index, CAIT: Cumberland ankle instability tool, ^#^ CAI vs. Coper, ^*^ CAI vs. Healthy, ^✝^ Coper vs. Healthy (all significant differences, *p*  <  0.05).

### Concurrent validity

In the force-matching test, significant correlations between the CON-TREX and the FF-PD devices were observed for both plantarflexion and dorsiflexion across all groups (Table 2). In the CAI group, moderate correlations were found for plantarflexion (*r*  =  0.608, *p*  =  0.002) and dorsiflexion (*r*  =  0.597, *p*  =  0.002). In contrast, strong correlations were observed in the coper group (plantarflexion: *r*  =  0.753; dorsiflexion: *r*  =  0.717; both *p*  <  0.001) and the healthy group (plantarflexion: *r*  =  0.770, *p*  <  0.001; dorsiflexion: *r*  =  0.627, *p*  =  0.001). When all participants were analyzed together, strong correlations were also observed for plantarflexion (*r*  =  0.724, *p*  <  0.001) and dorsiflexion (*r*  =  0.636, *p*  <  0.001).

**Table 2:** Concurrent validity of AE% between the CON-TREX and the FF-PD devices in the force-matching test.

Movement direction	Correlation between AE% of the CON-TREX and the FF-PD devices			
CAI	Coper	Healthy	Whole group
*r* (*p*-value)	*r* (*p*-value)	*r* (*p*-value)	*r* (*p*-value)
PF	0.608 (0.002)	0.753 (< 0.001)	0.770 (< 0.001)	0.724 (< 0.001)
DF	0.597 (0.002)	0.717 (< 0.001)	0.627 (0.001)	0.636 (< 0.001)

Notes: AE: Absolute error, *r*: Pearson correlation coefficient, PF: Plantarflexion, DF: Dorsiflexion.

### Discriminant validity

The one-way ANOVA revealed significant group differences in AE% for plantarflexion (*F*  =  5.099, *p*  =  0.01, Table 3) and dorsiflexion (*F*  =  3.604, *p*  =  0.037). Post hoc comparisons revealed that the CAI group exhibited a significantly greater AE% than the healthy group in both plantarflexion (*p*  =  0.011, Cohen’s *d*  =  0.88) and dorsiflexion (*p*  =  0.034, Cohen’s *d*  =  0.75), while no other significant group differences were observed (*p*  >  0.101). The optimal AE% cutoff for discriminating individuals with CAI from healthy controls was 12.06% for plantarflexion, with an area under the ROC curve (AUC) of 0.73 (95% CI: 0.58–0.87), sensitivity of 70.8%, and specificity of 75.0%. For dorsiflexion, the optimal cutoff was 9.57%, with an AUC of 0.70 (95% CI: 0.55–0.85), sensitivity of 87.5%, and specificity of 45.8%.

**Table 3:** Validity of the FF-PD device for group discrimination in the force-matching test.

Movement direction	Mean ± SD			One-way ANOVA	
CAI	Coper	Healthy	*F*	*p*-value
PF (AE%)	15.7 ± 6.9	13.2 ± 4.3	10.5 ± 4.5*	5.099	0.01
DF (AE%)	16.8 ± 6.9	14.6 ± 2.4	12.2 ± 5.2*	3.604	0.037

Notes: FF-PD: Fixed-frame portable dynamometer, PF: Plantarflexion, DF: Dorsiflexion, AE: Absolute error, ^*^ CAI vs. Healthy (significant differences, *p*  <  0.05).

### Test–retest inter-session reliability

The FF-PD device demonstrated good to excellent test–retest reliability for both plantarflexion and dorsiflexion across all groups (Table 4). For plantarflexion, ICCs ranged from 0.855 to 0.960, with an ICC of 0.891 (95% CI: 0.849–0.919) observed in the whole group, SEM values between 0.9% and 2.6%, and MDC_95_ values between 2.5% and 7.2%. For dorsiflexion, ICCs ranged from 0.794 to 0.886, with a whole-group ICC of 0.874 (95% CI: 0.823–0.910), SEM values between 1.2% and 2.7%, and MDC_95_ values between 3.4% and 7.4%.

**Table 4:** Test–retest reliability of the FF-PD device across two sessions in the force-matching test.

Group	Movement direction					
PF			DF		
ICC (95%CI)	SEM (AE%)	MDC_95_ (AE%)	ICC (95%CI)	SEM (AE%)	MDC_95_ (AE%)
CAI	0.855 (0.720–0.911)	2.6	7.2	0.886 (0.810–0.931)	2.7	7.4
Coper	0.855 (0.728–0.898)	2.1	5.8	0.794 (0.593–0.900)	1.2	3.4
Healthy	0.960 (0.905–0.977)	0.9	2.5	0.856 (0.732–0.917)	2.0	5.7
Whole group	0.891 (0.849–0.919)	2.0	5.6	0.874 (0.823–0.910)	2.1	5.8

Notes: PF: Plantarflexion, DF: Dorsiflexion, ICC: Intraclass correlation coefficient, SEM: Standard error of measurement, MDC_95_: Minimal detectable change (MDC) at the 95% confidence level, AE: Absolute error.

**Fig 3:**
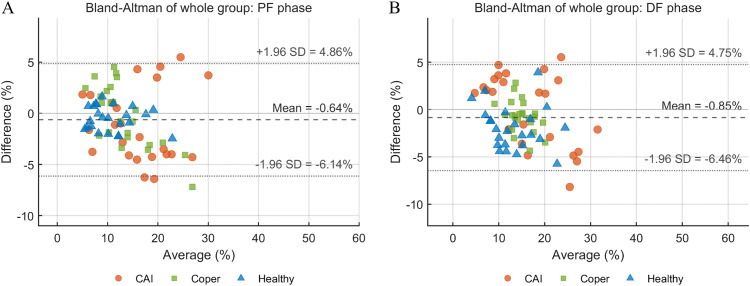
Bland–Altman plots illustrating the agreement of AE% between sessions in the force-matching test for (A) plantarflexion and (B) dorsiflexion. The long dashed line indicates the mean bias, and the short dashed lines represent the 95% limits of agreement (± 1.96 SD).

The Bland–Altman analysis of the overall sample revealed a mean bias of –0.64% (95% limits of agreement: –6.14% to 4.86%) for plantarflexion and –0.85% (95% limits of agreement: –6.46% to 4.75%) for dorsiflexion, indicating good agreement between sessions (Fig 3).

## Discussion

### Concurrent validity

The analysis of concurrent validity between FF-PD and CON-TREX demonstrated significant moderate-to-strong correlations across all groups, with stronger correlations observed in the coper and healthy groups. These findings indicate that the FF-PD can reliably assess force sense across different populations. However, the comparatively lower correlations in the CAI group indicate that the FF-PD may inadequately reflect the subtle variations in force sense among individuals with CAI. The heterogeneity of CAI may account for this limitation because some individuals displayed considerable deficits in proprioception, balance, and force control, whereas others present with milder impairments [14,30]. Moreover, individuals with CAI often develop compensatory strategies, such as modified sensory weighting, which can affect the precision of force-matching tests and thereby reduce the correlation between the CON-TREX and the FF-PD devices [8,31].

In addition, force-matching tests may be insufficiently sensitive to identify the nuanced elements of force sense, which are affected by intricate cortical and subcortical neural processes. Evidence from healthy individuals indicates that beta-band activity in the primary sensorimotor cortex is intricately associated with the precision of ankle force control [24]. This cortical activation pattern may be compromised in CAI, resulting in diminished accuracy of force control [9]. Future research should incorporate neurophysiological methods, including electroencephalography and electromyography, to investigate the interplay between cortical activity and muscular responses. This approach would yield a more thorough comprehension of the neurobiological foundation of force sense and improve the sensitivity of FF-PD in identifying deficits in CAI individuals.

### Discriminant validity

Significant differences in AE% among groups, especially between the CAI and healthy groups, substantiate the discriminant validity of the FF-PD. The CAI group exhibited significantly higher AE% in plantarflexion and dorsiflexion, suggesting that the FF-PD can effectively distinguish individuals with functional impairments from those with normal force sense. This finding aligns with previous research, confirming that individuals with CAI exhibit deficiencies in force sense relative to healthy controls [5]. From a clinical perspective, the FF-PD may be most appropriately applied in individuals with a history of ankle sprain or suspected CAI, particularly during early screening and longitudinal monitoring. Given its portability and acceptable discriminative ability, it may serve as a practical tool for identifying impaired ankle force sense and informing the need for further clinical evaluation or targeted rehabilitation. In addition, the good test–retest reliability and the magnitude of the MDC_95_ suggest that the FF-PD may be suitable for follow-up assessments and for evaluating treatment-related changes, provided that observed changes exceed measurement error. However, no significant differences were found between the coper group and either the CAI or healthy groups. This result can be attributed to the relatively minor deficits in force perception in copers and their adaptive balance recovery, leading to task performance akin to that of healthy individuals [18].

Moreover, the AE% cut-off values distinguishing individuals with CAI from healthy controls exceeded the corresponding MDC_95_ values for both plantarflexion and dorsiflexion, indicating that these thresholds surpass measurement error and therefore are likely to reflect clinically meaningful differences. This finding supports the use of these cut-offs as actionable thresholds for screening and clinical decision-making in real-world practice. However, the relatively low specificity observed for dorsiflexion in the ROC analysis warrants careful clinical interpretation. Lower specificity indicates an increased likelihood of false-positive classification, whereby some healthy individuals may be identified as impaired. Consequently, dorsiflexion force sense assessed by the FF-PD should not be used as a stand-alone diagnostic criterion. Instead, elevated dorsiflexion AE% values should be interpreted within a broader clinical context and followed by confirmatory assessment, such as patient-reported outcome measures, clinical instability tests, or additional sensorimotor evaluations. This stepwise approach may help mitigate the risk of inappropriate clinical decision-making while preserving the high sensitivity of the FF-PD as a screening-oriented tool. Subsequent research should further validate these cutoff values and investigate methods to improve specificity, thereby enhancing the diagnostic accuracy of the FF-PD.

With respect to the interpretation of force-matching performance, it should be noted that the present study quantified force sense using absolute error (AE%), which captures the magnitude of deviation from the target force but does not retain information on error direction (i.e., overshooting or undershooting). Consequently, the current results do not permit determination of whether individuals with CAI tended to exceed or fall short of the 25% MVIC target, nor whether error direction differed between CAI and healthy controls. Despite this limitation, AE% remains clinically relevant for assessing discriminant validity, as larger absolute errors reflect impaired force sense irrespective of error direction. From a screening perspective, the ability of the FF-PD to identify individuals with elevated force-matching error magnitude is valuable for detecting sensorimotor deficits associated with CAI. Future studies should incorporate signed error metrics alongside AE% to characterize error directionality, which may provide further insight into motor control strategies in CAI and support more targeted rehabilitation goal setting.

### Test–retest inter-session reliability

The test–retest findings indicate that the FF-PD generally demonstrated good-to-excellent reliability across groups, supporting its overall stability and suitability for repeated measurements. However, these results should be interpreted with some caution. Although ICC point estimates ranged from 0.794 to 0.960, the lower bound of the 95% CI for dorsiflexion force sense in the coper group reached 0.593, suggesting that reliability for this specific condition may fall within the moderate range. This finding highlights a degree of uncertainty in dorsiflexion force sense measurements among copers and underscores the importance of considering confidence intervals alongside ICC point estimates when interpreting reliability.

SEM values ranged from 0.9% to 2.6%, indicating acceptable measurement precision and supporting the FF-PD’s ability to detect relatively small changes in force sense. Correspondingly, MDC_95_ values (2.5%–7.4%) suggest that changes in AE% exceeding these thresholds are likely to reflect true physiological variation rather than measurement error. From a clinical perspective, this is particularly relevant for longitudinal monitoring and treatment evaluation, provided that observed changes surpass the MDC_95_. Bland–Altman analysis further demonstrated minimal inter-session bias, supporting acceptable agreement between sessions. Future studies should further investigate factors contributing to variability in dorsiflexion force sense reliability, particularly in coper populations, to refine testing protocols and enhance measurement stability in clinical and research applications.

Several limitations should be acknowledged. First, this study assessed only short-term test–retest reliability and did not examine long-term stability. As force sense may vary over time, particularly during rehabilitation, future studies should evaluate the long-term reliability of the FF-PD to support its use in longitudinal monitoring. Second, the assessment was restricted to sagittal-plane ankle movements, while inversion and eversion were not included. Given the relevance of multi-planar control to ankle instability, future research should extend FF-PD assessment to frontal-plane movements. Third, differences in testing body position between the computerized dynamometer and the FF-PD may have introduced subtle biomechanical variability that could affect inter-device correlations and group discrimination, although gravity effects on sagittal-plane ankle force are likely minimal due to the use of AE%. Future studies may mitigate this variability by further standardizing testing posture, such as with a wall-mounted FF-PD. Fourth, only right-leg dominant participants were included to reduce variability related to limb dominance, which may limit generalizability to left-leg dominant individuals. Bilateral testing should be considered in future studies. Finally, the sample consisted exclusively of young adults, which may limit applicability to other populations. Future research should include broader age ranges to confirm the clinical utility of the FF-PD across different demographic groups.

## Conclusion

FF-PD demonstrates good reliability and efficacy in assessing ankle plantarflexion and dorsiflexion force sense, effectively differentiating individuals with CAI from healthy controls owing to impaired force sense in CAI.
